# Introductory Lecture, Delivered before the Class of the Medical Department of the Washington University

**Published:** 1849-01

**Authors:** Thos. E. Bond

**Affiliations:** Professor of Therapeutics, Materia Medica and Hygiene; Nov. 11th, 1848.


### ARTICLE III.

*Introductory Lecture, delivered before the Class of the Medical Department of the Washington University.* By Thos. E. Bond, Jun., A. M., M. D., Professor of Therapeutics, Materia Medica and Hygiene; Nov. 11th, 1848.

Next to the task of preparing a king's speech from the throne, which, by those conversant with the scope and aim of those short but elaborate productions, is considered one of the severest tasks of intellect, I know of nothing more difficult than the preparation of an introductory lecture to a course of scientific instruction, to be read to a class of students, in the presence of an intelligent miscellaneous audience.

Difficult as it is at all times and to all men, it is particularly so *now* and to *me*. Having had the pleasure to *hear*, I have now the misfortune to follow, a series of excellent lectures, pronounced by able and eloquent men; any of whom it is disadvantageous to succeed, as it would be presumptuous to hope to excel.

Those of you who were here last night will *sympathize* with *me*, if you have any bowels of compassion. Together with myself, you listened to one of the ablest and most eloquent introductory lectures perhaps ever pronounced before a medical class: a lecture which, for propriety of conception, elegance of diction, clearness of expression and force of utterance, will bear favorable comparison with the best of its kind, and which has at once placed my friend who delivered it, among the very foremost of the medical teachers in the country. Such is the man whom I must follow; and while the music of his words is yet singing in your hearts, and his happy pictures dancing before your eyes, I must intrude an address upon you, which, though it may not be compared, yet will not fail to be contrasted with the remarkable performance which immediately preceded it.

In the city of New York, I once met a gentleman and his wife, who had left their home in a small village of this state, to visit the great metropolis. Intent upon seeing sights, they found their way to one of those gorgeous churches, which, in that splendid city, so amaze the observer unaccustomed to architectural magnificence. Together they stood beneath the noble dome, so light, so airy, so beautiful, so chaste, it seemed a canopy let down from heaven, to cover the sacred edifice it hardly appeared to touch. In silent wonder they paced the shadowy aisles, beyond which stretched an endless colonnade, whose chiseled shafts and polished capitals were bathed in the soft prismatic light, which streamed through golden and manytinted windows—beautiful emblem of that ray of truth which, falling from heaven on the church below, is refracted into all the hues which constitute the bow of promise.

Wonder, awe and admiration for awhile were too strong for words; but at length these powerful emotions found utterance, and the stranger exclaimed, "Polly, Polly, when we go home, we must whitewash the meeting-house!"

Somewhat similar were my own feelings after hearing the admirable lecture of my reverend and learned friend. I thought I must go home and put some better appearance upon what I had prepared for you. But fortunately I remembered the fable which warns of the consequence of inflating one's-self beyond the natural dimensions.

In truth, gentlemen, there is room in the world for the humble meeting-house as well as the gorgeous temple. Its homely accommodations are, nevertheless, often gratefully appreciated; and whether whitewashed or not, its evident unpretendingness will secure it from criticism.

In endeavoring to select some subject, the discussion of which might be agreeable to you, and at the same time not uninteresting to the mixed audience who have honored us with their presence this evening, it has been difficult to resist the temptation to prepare an introductory more amusing than useful.

There are many topics incidentally connected with medicine, which afford abundant material for such an entertainment; and of these it would have been easy to construct a discourse, pleasant as comedy or ludicrous as farce.

But, gentlemen, we are met on business, and business too of a sober kind. To-night introduces you to the study of a most extensive and highly important branch of medical science, upon your proficiency in which will greatly depend your future success in the most difficult and delicate of all pursuits; and it would be unjust to you to waste such an opportunity for giving impulse and direction to the efforts you are about to make to acquire correct and varied information. I will endeavor, therefore, to offer you some practical suggestions upon your course of study, having chosen my subject, as Mrs. Primrose did her wedding gown, "not for a fine, glossy surface, but for such qualities as she thought would wear well."

In the first place, then, it is all-important that you shall have chosen your future profession thoughtfully, and with due consideration of its nature and consequences.

In days of yore, our forefathers had knowledge of an extraordinary country, called the land of Cockgaine, where it rained hot custards three times a week, and where all other good things might be had upon the easy condition of *sleeping* for them. Unfortunately for the indolent of modern days, the historians of that land of ease and gastronomic beatitude neglected to record the geography of it, so that the recollections of its walls of sausage, roofs of lard, and laths of barley sugar, are among those memories of joys that are past, which are sweet and mournful to the soul.

Yet men will have their lands of Cockgaine: their happy vallies: their fairy regions, where subsistence may be sure without effort, and where life shall be passed as one long summer's day of indolence and ease.

Now it is barely possible that human folly and youthful inexperience may sometimes err so far as to locate this ideal Paradise within the realms of Esculapius, and conceive the Profession of Medicine the Το Αγαθον of the luxurious and the lazy. It is but frank to say, that if any one of you has come here under the impulse of so mad a fancy, he has sadly mistaken the road to Cockgaine; he must seek hot custards and barley sugar by some path which we have not been fortunate enough to find.

Medicine is a science: perhaps of all sciences the most intellectual, for it gives full employment to every faculty of the mind. It is a benevolence which calls for all that is pure in morals, generous and heroic in conduct: which gives opportunity for the nearest possible approach to the example of "*Him* who went about doing good."

To the brutish man the study of medicine has no charms; the practice of it no enjoyments. None but the noble and the intelligent are worthy of the honors, as none but they are capable of the pleasures of the profession. To them, indeed, it offers inducements stronger than promises of ease; more alluring than dreams of luxury. To minds like these, we can conceive of no pursuit more congenial. Leading the student into the widest and most fruitful fields of nature, it spreads before him the curious things of the mineral, vegetable and animal kingdoms; then throws wide the portals of the moral and intellectual worlds, and bids him seek, in the magnificent developments of mind and soul, those wonderful laws of being which unite the sentient and corporeal man. It is his glorious privilege both to learn and discover truth; not merely to receive the dogmas of a completed science, but to examine for himself the innumerable facts and observations of well-spent centuries; to deduce from them laws of action and relation, and to add to the vast common stock whatever he may discover of new, or ascertain of the previously doubtful. In every stage of his progress, whether in study or practice—whether in his closet he learns the history of disease and power of remedies, or whether he resolutely bends over the putrid relics of decayed mortality; or whether, by the bedside of the pestilential sick, he boldly traces symptoms and distinguishes disorders—everywhere, under all circumstances, he is seeking *truth;* stripping it of disguises; searching it out from the midst of false plausibilities; discriminating it from deceitful errors.

He seeks *truth:* not that it may be his own, but that he may give it to the world; for the vows of his profession are upon him, and he may not treasure for himself the secrets of an art which might be cheaply bought for thousands of gold and silver. He finds himself enrolled as a neophyte of a profession more ancient than story; as durable as man: a profession whose records are an unbroken chronicle of the achievements of the intellectual few, for the good of the suffering many; a profession sung by poets, revered by sages, and honored by the daily homage of the human race. Unrolled before him lays the recorded experience of ages; the accumulated gains of mental labor, as intense and honest as was ever expended upon the phenomena and laws of nature. There he may trace the progress of intellect through its long and fearful struggle with ignorance and error. He may follow the way of truth from its early twilight, until *intellect blazes* in its strength, and innumerable minds catch the golden radiance and kindle into light. He feels that he may be a partner of all this glory; he too may be a star in the intellectual firmament; nay, a full orbed *sun*, shining in his own strength, the centre of his own system.

If the study of medicine be ennobling, the practical duties of the profession are certainly not less so. Benevolence, the accident of others, is the business of the physician. His daily errand among men is to heal the sick, to assuage pain, to comfort the sorrow-stricken. To him, and only to him, is it given to "stand between the living and the dead and stay the plague." To him, and only to him, is it given to dispense health to the sick, and beauty to the deformed, and call back wandering reason to her deserted throne! Though his power be limited, yet is it power the most magnificent ever won by the intellect of man. True, the abundance of medical aid, and the freeness with which it is offered, has lessened its nominal value to the thoughtless, and so, too, the abundance of the rill and river make men unmindful of the treasure of water, which gushes profusely at their feet. But let the heavens be shut, and the streams fail, and priceless becomes the diminished store of that precious fluid, but now so little prized. So, gentlemen, were medical skill to perish from the earth, until but one physician remained, and he the least skilful of all who have deserved the name, he would be to men the most valuable of all possessions; his life would be precious, far beyond the life of kings, and the worth of his aid would be measured only by the value of ease to the agonized, and of life to the dying.

But the student having deliberately determined to devote himself to medical study, and having formed a proper conception of the character and conditions of the profession, how may he best attain his object?

The first requisite for success is a fair degree of *bodily vigor.* Of course we would not measure the capacity of a pupil by the breadth of his shoulders or size of his limbs. Doubtless many an excellent grenadier would make but a sorry physician.

But the study of medicine, as all intellectual pursuits, requires sound health, to withstand the wearing of the active mind upon the comparatively quiescent body; while the harassing duties of the active physician are often too severe for even the strongest frame. In no employment is it more important to possess the "mens sana in corpore sano." Perhaps there is none from which so many are compelled to retire, through sad experience of their physical inability to perform its arduous duties. With the exception of a few who inherit the fatal legacy of pulmonary tubercle, our medical classes are mainly composed of robust, vigorous young men, chiefly drawn from the country, and free from the constitutional debility which too often characterizes the children of the city. Yet, before the close of their probationary studies, it is not uncommon for some of them to break down so utterly, that the commencement day, so ardently anticipated, finds them panting on the top of Pisgah, from whose toil-won summit they look upon the promised land, they have not strength to enter and possess; or should they make a lodgment there, a few years of professional labor consigns them to the tomb, or drives them, infirm and broken-spirited, to other and less laborious pursuits.

The cause of this waste of health and life is a serious question to you and to society. Is it intrinsic to medical pursuits, or due to accidental and unnecessary causes?

That there is nothing in the study of medicine, in any of its branches, however apparently insalubrious, that is prejudicial to health, is an opinion sustained by an array of facts altogether irresistible; and it is unnecessary at present to justify it by argument. We must therefore seek the cause of the evil in some accidental and unnecessary circumstances, which, nevertheless, are of common occurrence to medical students. The obvious desire to be useful to you, must excuse the plainness with which it will be necessary to indicate some prominent errors through which, too often, the health of the student is sapped, and his prospects irremediably blighted.

One cause of the evil in question is, undoubtedly, to be found in what is called dissipation, in all its degrees, from mere irregularity to excessive profligacy.

A great city is, in many respects, a great evil. It abounds with temptations to pleasure, and the inexperienced young man is easily induced to take a first downward step, which frequently plunges him into a vortex of vicious indulgence, from which, if he ever escapes at all, he emerges shorn of his strength, corrupt in body, mind and soul.

It is not necessary to dwell upon this unpleasant theme. To the wise, a word of warning is sufficient; to the inconsiderate fool, all argument would be vain.

I would affectionately call your attention to another error, almost as dangerous as the one already named. It consists in an excessive and irrational application to study.

In every medical class are found young men of ardent temperaments, who begin their studies with an earnestness amounting to enthusiasm, and concentrating all their energies upon the pursuit of professional knowledge, overtask mind and body, and entail upon themselves premature infirmity, if not sudden and untimely death.

The consequences of this ill-advised and inconsiderate zeal are the more deplorable, that the victims of it are those whose natural endowments and untiring industry give most pleasing promise of eminence in their profession and usefulness to the public.

In many a forgotten grave there lies, cold and still, a young heart, which lately throbbed as yours is throbbing; a heart whose epitaph might well be written in the beautiful lines of Byron:

"Oh! what a noble heart was here undone!
When Science' self destroyed her favorite son!
Yes, she too much indulged thy fond pursuit:
She sowed the seeds, but death has reaped the fruit.
'Twas thine own genius gave the fatal blow,
And help'd to plant the wound that laid thee low.
So the struck eagle, stretched upon the plain,
No more through rolling clouds to soar again,
Viewed his own feather on the fatal dart,
And winged the shaft that quiver'd in his heart.
Keen were his pangs; but keener far to feel
He nursed the pinion which impell'd the steel,
While the same plumage that had warmed his nest,
Drank the last life-drop of his bleeding breast."

Be assured that nothing can be gained by violating immutable physical laws. *He* will study to most advantage, who will carefully estimate his powers of attention and endurance, and resolutely refrain from tasking them unduly. The student who sits up until midnight to pore over a book which no longer interests him, and who rises at early dawn to renew the irksome task, will certainly read a great deal during the winter, and just as certainly will learn but little. The sustenance of the mind, as of the body, depends less upon quantity of food than completeness of digestion. The excessive feeder is always badly fed.

As a general rule, it is not profitable to read when the attention can no longer be fixed without painful and repeated efforts. It is better, in such cases, to relieve the wearied mind by some less irksome study or rational recreation. Of course it is not to be understood that a student should not read except when vehemently inclined to it.

Some young gentlemen are endowed with an instinctive prudence in respect to excessive study, which relieves their preceptors of all anxiety about their health, so far as danger from that error is concerned. Were these never to read until the yearning for knowledge became too powerful to resist, a whole session would hardly suffice for the accomplishment of a course of study more extensive than that comprised in Patrick Henry's library, which was said to have consisted of Blackstone's commentaries, Shakespeare's plays, a bottle of brandy and a fiddle; and the end of their college terms would find them in possession of an amount of medical knowledge, second in extent and accuracy only to that of Dame Quickly, who described Falstaff's fatal sickness as a "burning quotidian tertian."

Such minds can only be brought to the effort and protracted labor of study by the exercise of a resolute will, and the unmitigated control of a well devised system and method of attention.

The student should always allow himself an hour or two of the evening for social intercourse and agreeable conversation. This will do him more good than public shows, or even those preposterous, ill regulated entertainments, called evening parties; though, in venturing to caution you against the latter, I know I am arraying myself against fascinations which are irresistible, and which I would by no means wish you to resist. If you cannot have the society of ladies without going out at bed-time, standing upon your reluctant legs till morning, and devouring enormous quantities of incongruous dainties: mingling in one tired and sleepy reservoir, oysters and ices, pound-cake and poultry; if this must be the price of ladies' society, then go—for the society of ladies you must have, if you would retain the civilization you have brought with you from the homes of your mothers and your sisters. But avoid the alternative if you can. Rather content yourselves with the society of ladies who are older if not so pretty. You will find them better company and truer friends. An hour's chat with a sensible woman of sixty, will do you vastly more good than two hours' dancing with a lively gipsy of sixteen. This arrangement will be the more advisable, that it will secure you against the danger of falling in love during the session—a danger of all others to be dreaded. Other things are evils; but this is utter calamity—the very verge of catastrophe itself.

Many students, finding themselves unable to get through what they suppose to be the necessary amount of reading during the six days allotted to man for labor, are induced, upon the common pretence of necessity, to desecrate the day of rest by continuous study.

Now, gentlemen, it is not my province to vindicate the sanctity of the Christian Sabbath as a theological dogma; but as a hygienic regulation of the Creator Himself, I feel bound to contend for the intellectual and physical advantages of its observance. The appointment of this septdiurnal rest is intimately related to the laws of man's nature, and it is as absurd to disregard this Divine prescription, as it would be to violate the law of our physical frame which demands that the exhausted body shall be restored by sleep.

It matters not the enforcement of the precept is in the one case committed to an involuntary instinct, while in the other it is confided to the judgment of a moral and intelligent being. The injunction is equally positive, and similarly founded upon the immutable laws of our constitutional existence. The man who disregards the wise and benevolent provision made for his reinvigoration, assumes to understand his wants better than they are understood by the God who made him, and is justly liable to whatever of penalty naturally attaches to such presumption.

No man will find himself wiser for his inappropriate Sunday studies. It will be well for him, if dull perception, weakened judgment and treacherous memory do not soon appear in evidence against the recklessness with which the mind has been overworked and exhausted.

It is not necessary to dwell upon this subject, but experience and observation tell us that it is not irrelevant to suggest it to you. Unfortunately, the circumstances of a physician's life too easily lead to forgetfulness of the Sabbatical observance in question, and the influence of Parisian education, however beneficial in other respects, has doubtless strengthened the tendency. It has even become fashionable to affect a certain superiority to public opinion upon this subject. Sunday has been made the operation day in hospitals, and the student has been encouraged to shake off the habit of his father's house, and assume the professional independence of a man who is hereafter to consider science as the chief good, and the pursuit of it to involve exemption from all inconvenient moral restraints. An error so gross can produce only evil—physical, moral and intellectual; and we would not be acting humanely did we not warn you against it.

It is very important that the student should commence his studies with a correct comprehension of the nature and end of medical science: a subject upon which there exists a great deal of error among writers, teachers and physicians; and error too of the most dangerous practical character.

The whole world seems suddenly smitten with a passion for *Philosophy*, or at least for the word, for as to the thing itself, there is no excess to complain of. We cannot open a child's school book without being stared in the face by this formidable Greek word; indeed, it is with difficulty one can escape having his breeches made upon philosophical principles. The bench philosophizes on law; the pulpit on religion; the physician on medicine. The farmer ploughs a philosophic furrow; the barber flourishes a razor sharpened on philosophic principles; and the dancer cuts a pigeon-wing according to the established laws of the philosophy of motion. In short, we may almost adopt the language of Jarvis in the comedy of the Good Natured Man, who said, that "whenever he heard his master mention Philosophy, he was sure he was going to play the fool."

The truth is, the word has almost lost its original signification, and could it lose it entirely, the use of it might cease to be dangerous. As it is, the term serves to confound things essentially different; and what is ostentatiously paraded as philosophy, is often, of all things, the most unphilosophical.

Gentlemen, medical science has nothing whatever to do with *metaphysics*, in the proper sense of that very familiar, but not very intelligible term. Medicine has nothing to do with abstractions or violent presumptions. It is eminently and exclusively a science of *phenomena* or *facts*. It does not, like philosophy, strictly so called, aspire to the comprehension of *essences* or *final causes*. It contents itself with the observation and knowledge of laws. Metaphysics speculates as to the ultimate nature of things in themselves, apart from sensible appearances, and has been well described as the art of methodically bewildering one's-self. Medicine, as all physical science, confines itself to the constant succession of phenomena, and generalizes them into laws.

Thus the medical philosopher or metaphysician speculates upon the essential nature of *life*. He would compass an idea of the vital principle, and from this deduce the rationale of phenomena and rules for medical practice. But all such and similar attempts have been and must always be vain. Life, abstractedly, cannot be investigated; it cannot be analyzed; it cannot be separated from its sensible phenomena; it cannot be studied; and to practice upon *notions* of it, is to presume to instinctive knowledge and intuitive comprehension of what cannot be comprehended.

"Optics good it needs, I ween,
To see what is not to be seen."

People unlearned in these matters, have an idea that *Philosophy* is identical with *Truth:* whereas, in whatever way applied, abstract philosophy has always been *false*, however brilliant and ingenious its theories, or honest the authors of them. From Plato to Samuel Thomson the steam doctor, all have based their doctrines on false assumptions.

It causes a smile, to class the ignorant and conceited clown with the master spirit of the Grecian schools; but however inferior in mental endowments Thomson was to Plato, and however vulgar and gross his system, yet it was, as to its nature, purely philosophical. It was based upon a presumed knowledge of essences, and the practice followed upon the a priori assumption as logical deduction, experience being merely a corroborative of the truth. Thomson assumed that *heat* was *life*—and *cold*, *death;* and therefore he stewed his miserable patients, in defiance of all the results of medical experience, and in spite of the phenomena which continually contradicted his confident predictions. After all, this poor man's theory, if more simple than those of learned doctors, was quite as true, or to speak more correctly, no more false.

Dr. Rush used to say, that "science was an eel, and forming theories was taking it by the tail." Unfortunately, he made the attempt himself, and the subtile thing slipped through his fingers as readily as it had done from the less nervous grasp of his predecessors. A disappointment which might have taught the hopelessness of the task, for who may hope to do what Rush could not?

The genteel Lilliputian quackery of our day, which bears the imposing name of homœopathy, is another example of the utter absurdity of rejecting all experience and common sense for the sake of a baseless theory: a system which, as the sailors say of a ricketty ship, is only kept together by its paint.

Homœopathy is a thing of words without ideas; a wild dream of medical indigestion, without coherence of parts, or basis of fact. It is unreality set to practice nothingness.

As the so-called principles of this fashionable medical philosophy may not be known to you, I will quote one or two of the primary dogmas; which might be called fundamental, only that they themselves have no foundation. Upon them, however, is built whatever of the system can be said to have any basis at all; for a great deal, especially what must by courtesy be considered the practical part of it, is such a farrago of absurdities as to defy all detection of the mental process by which they were conceived. It is impossible to imagine how they got into any unfurnished head, and equally difficult to guess why they should have been let loose when detected there by the owner.

It seems, however, to be ordained, in order to man's intellectual probation, that there should always be in every community some means of distinguishing people who do not think from those that do. Sydney Smith said that the corn laws question was the test in England. We have many here, and homœopathy is one of them. The great leading principle of Hahnneman is, that "to the entire human organization is superadded an immaterial principle—a *dynamical* or moving force—active in itself, by which the organization is ruled and controlled. It is this dynamical or ruling force or principle, upon which all morbific causes or influences act; and the disturbance which these causes occasion in this principle, operates of necessity upon the organization, deranging its healthy actions, and perverting its natural sensations."

Now here is stated a doctrine of the most startling character, which, if true, at once removes medicine from the physical to the metaphysical sciences. The seat of disease is here declared to be beyond the "organization," that is, beyond the body. The man proper is "superadded." But how can anything which has no materiality be added or subtracted either? These are mathematical terms, and cannot be applied to things without sensible qualities, and without defined relations.

Upon what evidence are we asked to believe this monstrous proposition? Why simply upon the ipse dixit of Hahnneman! Indeed, no evidence could be brought to bear upon it. Being beyond the material world, it cannot be investigated or proved. If known at all, it must be by authoritative revelation.

Leaving the very existence of this essential force entirely unproved, Hahnneman proceeds to declare the laws of its action, with as much assurance as though they had been revealed from on high. He says, "Every modification of this immaterial and independent principle manifests itself by external signs and symptoms, as altered actions and deranged sensations of organs. The changes themselves are beyond the reach of investigation."

So, then, it appears that immateriality is the only thing that matter can morbidly affect. It seems, too, that this thing, which cannot be investigated, can, nevertheless, be *modified:* to be *modified* is to be altered in its modes; but how can that be *changed* which cannot be observed. It seems, too, that disordered sensation depends upon an alteration in the immaterial superaddition or unorganized man, and not in his parts. If you bring an acid in contact with an exposed nerve of a tooth, the injury inflicted is not upon the sensitive part, but upon the immateriality, which being thereby changed, not chemically, of course, but homœopathically, moves the tooth to ache, by way of giving information. The doctor, coming to your aid, does not waste time upon the suffering organ itself, but addresses his remedy to the injured immateriality, and prescribes a specific, ascertained by a process similarly felicitous and unintelligible to that by which the immateriality itself was discovered. Except, indeed, when the doubting disciple of this shadowy school slyly substitutes a good round dose of morphia for the orthodox prescription.

It is not my intention to occupy your attention with an elaborate exposure of the queer details of homœopathic practice.

It might shock the delicate "immateriality" of some fair lady present, to be told that her elegant parlor sickness, which became her so well, and which yielded to four months' daily visitation on the part of her philosophic physician, was considered by him, and duly registered in his book of cases, as none other than the *itch;* and it might shake the confidence of some really disordered man, who is staking health and life upon the very rational prescriptions of Hahnneman, to learn that the potential little pill which he fingers with so much reverence, and swallows with so much hope, is in fact composed of exactly as much brimstone as would balance a moonbeam in the scales.

To the student who is curious to see the principles of this singular system discussed, I would recommend the very able and useful work of Dr. Bartlett, on the Philosophy of Medical Science; a work which ought to be carefully studied by every one who wishes to acquire correct views of the nature and end of medical inquiry.

That speculation of the wildest kind has contributed to the enlargement of medical science, is certainly true; but, as in the case of the positive sciences, all the aid thus afforded has been accidental.

The speculative theist found some imaginary relation between the bowels of a slaughtered beast and the secrets of the invisible world: while the astrologer traced a similar connection with the stars of heaven. Both were false and idle dreamers; yet the auguries of the one laid the foundation of physiology; and the horoscopes of the other led the way to those vast astronomical discoveries which are daily astounding the world. Each did mankind good service; but the benefit was accidental, while the evil was direct.

It is certainly true, that we know how to cure many diseases, and to mitigate all that flesh is heir to; but we do not know the ultimate cause or essential nature of a single one of them. Nor is it of the least consequence that we should. Such knowledge, of itself, would not increase our means of cure; it would not, in short, be any positive contribution to medical science.

A great deal of wit, and of what is intended for wit, is daily expended upon this supposed weak point in medicine. It is said to be a conjectural art, not worthy the name of science. We have heard this said by gentlemen of the bar, when contending for the superior dignity of their own science: one, by the way, which, noble, intellectual and honorable as it is, yet glories in uncertainty, and only escapes the reprobation of being *conjectural*, because no man who goes to law can reasonably *conjecture* when or how he shall get out of it.

The truth is, that medicine is but the result of long continued and sagacious observation of the phenomena of the living body and its relations, strengthened, corroborated, and in some small degree explained by microscopic examinations and the fruits of collateral physical inquiries.

This observation has furnished us with a great number of incontestable facts, which are as certainly ascertained as any other facts known to mankind. It has also fusnished us with a number of probabilities, which must yet be more fully tested before they can be considered absolutely true and certain. To learn these facts, to discern these phenomena, and to acquire skill to avail ourselves of our knowledge of their order and relation, so as to preserve health and cure disease: this it is to study medicine.

Hypotheses or theories have no necessary connection with medical science. So far as they embody the results of experience, they are well; but to base practice upon them, is building the pyramid upon its apex. They are prettinesses of medicine, and are harmless when kept out of the sick chamber. But medicine is in no way responible for them; their success adds nothing to our means of cure; their overthrow in no way concerns the stability of medical experience. Phenomena remain the same, and we practice upon them with equal certainty, whether they be best explained by Rush or Broussais, Hahneman or Thomson, or whether they all have failed to discover the truth. The planets kept their places in the heavens as securely under Ptolemy as Copernicus. Day and night came and went as regularly when the earth was thought to be a plane; and the physician draws upon established truths of experience as safely and successfully under the reign of one hypothesis as another.

We do not object to theorising. In itself it is a harmless and intellectual amusement. It is more dignified than playing billiards, and does not lead to bad company; but we do protest against mingling it with practical medicine, with which it has nothing whatever to do.

If medicine be not a *metaphysical*, neither is it a *mathematical* science. Mathematical deduction proceeds from a thorough and positive knowledge of elementary truths, upon which depend other truths as a necessary consequence; and thus the mind proceeds from the most simple, self-evident propositions to the solution of complicated problems. To make medicine a mathematical science, we must begin with a perfect knowledge of the elementary tissues of the body as vitalized things. We must drop the investigation of phenomena, and direct our attention to the essential qualities upon which they depend. We must take up the brain; find out not how it is, but what it is, and show why it necessarily perceives and thinks. We must take each article of the materia medica to pieces; find out why their effects are produced, and demonstrate the necessity of them. We must show why ipecacuanha vomits; why opium narcotizes—proving the necessity by the necessary dependence of things.

It is needless to say that this mode of procedure is impossible. None but the Great Creator himself can have perfect and complete knowledge of His works.

But is medicine, therefore, an uncertain science? If there be no truths but those that are mathematical, it is; but such is by no means the case.

It is certainly true that there exists in a beef-steak a nutritive quality capable of sustaining human life; but we have not ascertained the fact either by philosophy or mathematics. No a priori reasoning upon abstract principles could have developed such a fact, and certainly it was not reached by proceeding from an elementary knowledge of man and beef to the solution of the complex question of digestion. Yet the fact is true, and every body believes it.

Mathematics has to do with quantities; but experience teaches the qualities of things. Thus it was ascertained by accident or experiment that ipecacuanha has the quality to induce vomiting; opium the quality to induce sleep; and all our medical knowledge consists of separate isolated facts, which have no necessary dependence or connection. Opium does not narcotize because ipecacuanha vomits. There is no relation between the two truths.

Too much stress can hardly be laid upon this point, that medicine is composed altogether of a multitude of *experiences*, and that to experience we are to look for the improvement and perfection of the science.

There is a strong disposition to make medicine demonstrative and to give it as far as possible the air of an exact science.

The student is told that he must seek fundamental principles of practice in the necroscopic examination of the dead, instead of observation of the sick.

He is told that pathology, *i. e.* a knowledge of the sensible changes produced by disease upon the appearance of parts, is the foundation of medical science, and he is encouraged to devote to this part of study a very undue proportion of his time.

There never was a more absurd doctrine than this. So far is it from true that pathology is the foundation of medicine, that we are in the daily habit of curing diseases, of whose pathology we know nothing at all, and probably never will know anything. No one pretends to understand the pathology of intermittent fever, yet every tyro in medicine knows how it can be cured. Indeed the whole great class of fevers, is equally unintelligible in essential pathology, and nervous diseases hardly less so.

Even where pathology has won the greenest laurels, it has neverbeen able to do more than exhibit secondary consequences. The pathologist may point us to certain alterations of tissue and loss of parts in the lungs and tell us that herein lies the cause of all those indomitable symptoms which we call pulmonary consumption, but it will instantly occur to a thinking man that these changes themselves are but results, that preceding these were other manifestations of disorder, and that after all the pathologist has only succeeded in bringing to light another set of symptoms of the primary essential, and because essential, inscrutable disease.

If the disciple of the pathological school, determined on reaching the ultimate truth, should seize a microscope and push his inquiries into the delicate tracery of tissues inscrutable by unaided vision, and should he show you tiny vessels ruptured, reddened or enlarged, or some globules or atoms of morbid origin, you again, if you think at all, are compelled to ask whether these changes had no cause in prior changes, whether a microscope yet more powerful might not show that these conditions were but consequences.

In fact the pathologist will have followed up the chain of effects through another link, but the end, the primary point of attachment upon which all depend, will be as far off as ever.

Pathology is and must always be imperfect, it is and must always be a science of consequences and however curious, interesting and useful it may be, it is impossible to make it any thing more than an important accessory to medical science.

For many years it has been the darling pursuit of the ablest and most industrious men in the profession. Teachers, writers, painters, modelists and pupils have vied with each other in their zeal for this kind of investigation. The human body has been inspected with a minuteness and particularity, which could not be exceeded. The most powerful microscopes have been used to pursue inquires far beyond the limits of ordinary observation; and now how much are we the better able to cure disease for all this labor? Has it added one solitary medicine to our materia medica; or has it taught us to cure one single disease hitherto incurable? Is not experience, and only experience, the guide of every judicious and successful practitioner of medicine? Does it not remain true, that many of these diseases with whose pathology we are best acquainted, are precisely those which we cannot control, while others of whose *patholgy* we know little or nothing at all, we are in the habit of curing every day?

The common triumphant reply to these strictures is, that to know what is the matter, is the first step to correct treatment. The fallacy of the defence lies in the assumption that necrotic pathology displays the actual disorder, while in fact it merely traces its progress. It confounds certain facts in the history of disease with disease itself.

I am well aware that these remarks will fall strangely upon the ear. They are counter to the fashionable doctrines of the day, and the man who ventures to dissent from commonly received opinions must expect to be misunderstood, misrepresented and even defamed. It is as dangerous to be before that age as behind it. But this is nothing to the purpose. Truth must be served at all costs; to suffer in its cause is its own recompense.

Do not, however, understand me to say, that pathological study is useless. On the contrary it is useful, but it is by no means worthy of the rank which has been assumed for it in comparison with other medical studies.

It is full time, that our country had its own school of medicine. Dr. W. Holmes, characterizes national medical peculiarities, by saying that the Frenchman, in studying medicine, inquires, what is to *see* about it, the German what to *think* about it, and the American what to *do* about it. It is our purpose, gentlemen, to found here a school upon the "*do* about it" principle: an *American* school, to which France, Germany and England may contribute, but which neither nor all of them shall control.

To do this, we must encounter much of opposition. The struggle for success may be long; certainly it will be arduous. The teaching of medicine is mainly in the hands of men who have never practiced extensively; many of them not at all until their elevation to a practical chair gave them factitious reputation. Talents and learning and position and fashion give to such persons great influence, and it will not be easily shaken. But we have counted the cost, we have enlisted for the war, an American school we will have; and once established, imported error will go down before it as Dagon fell before the ark.

To our competitors in other cities, we wish no harm. May their shadows never be less! If they will unite with us to infuse a healthy national tone into American physic, we will rejoice in their success: if they will not, our respect for men shall not prevent us from attacking the errors to which they are wedded.

To our more immediate competitor, we wish a long career of increased and increasing prosperity. It would ill-become me, who have been honored with three several degrees from that University, to say anything discreditable of it. With its professors and with its students, we would cultivate the most friendly relations. Unless universal experience should fail, our success must contribute largely to their own. On the other hand, it will as little become our alma mater to disparage us. If we do not understand our profession, the community will cry shame equally upon us and upon those who gave us our titles and recommended us to the public.

We have undertaken this enterprise without the aid of the wealthy or patronage of the great. We have taxed our own slender means to the utmost, to rear a building creditable to our city, and comfortable to you. We have stored it with apparatus of the best and costliest kind, and we throw ourselves for success upon the intelligence of society, upon the good sense of the students, upon the kindness of the profession and upon that generous justice of our countrymen, which never yet has suffered enterprise in a good cause to be unrewarded.

The result we will abide in patience.—'Tis not in mortals to command success, we will do more: by trying to deserve it.

